# Supplementary material for: Recruitment dynamics of ESCRT-III and Vps4 to endosomes and implications for reverse membrane budding
Source: eLife. 2017 Oct 11;6:e31652. doi: 10.7554/eLife.31652 (PMC5665648; doi:10.7554/eLife.31652)
Supplement: Supplementary file 1. [file elife-31652-supp1.docx]

Supplementary file 1

| Statistics of the CLEM dataset |  | Vps4-eGFP | Vps4-mNeonGreen |
| --- | --- | --- | --- |
| Cells studied | 31 | 10 | 21 |
| Number of spots | 38 | 12 | 26 |
| Cells without spots | 4 | 2 | 2 |
| MVB clusters without signal | 11 | 3 | 8 |
| MVBs in those | 14 | 4 | 10 |
| Complete MVBs | 2 | 1 | 1 |
| MVB clusters with signal | 36 | 11 | 25 |
| MVBs in those | 83 | 26 | 57 |
| Complete MVBs | 15 | 8 | 7 |
| Clusters associated with sometimes more than one organelle |  |  |  |
| vacuoles | 42 | 13 | 29 |
| mitochondria | 2 | 1 | 1 |
| other organelles | 15 | 7 | 8 |
| Total clusters | 47 | 14 | 33 |
| Total MVBs | 97 | 30 | 67 |
| Total complete | 17 | 9 | 8 |
| Mean MVBs per cluster | 2,1 | 2,1 | 2 |
